# Deep sequencing of the uterine immune response to bacteria during the equine oestrous cycle

**DOI:** 10.1186/s12864-015-2139-3

**Published:** 2015-11-14

**Authors:** Christina D. Marth, Neil D. Young, Lisa Y. Glenton, Drew M. Noden, Glenn F. Browning, Natali Krekeler

**Affiliations:** Asia-Pacific Centre for Animal Health, Faculty of Veterinary and Agricultural Sciences, The University of Melbourne, 250 Princes Highway, Werribee, VIC 3030 Australia; Department of Biomedical Sciences, College of Veterinary Medicine, Cornell University, Ithaca, NY 14853-6401 USA

**Keywords:** Infectious endometritis, Oestrous cycle, Escherichia coli, Equine uterus, RNA-Seq, Next-generation sequencing, Gene expression, Ovarian hormones, Innate immune response, Transcriptomics

## Abstract

**Background:**

The steroid hormone environment in healthy horses seems to have a significant impact on the efficiency of their uterine immune response. The objective of this study was to characterize the changes in gene expression in the equine endometrium in response to the introduction of bacterial pathogens and the influence of steroid hormone concentrations on this expression.

**Methods:**

Endometrial biopsies were collected from five horses before and 3 h after the inoculation of *Escherichia coli* once in oestrus (follicle >35 mm in diameter) and once in dioestrus (5 days after ovulation) and analysed using high-throughput RNA sequencing techniques (RNA-Seq).

**Results:**

Comparison between time points revealed that 2422 genes were expressed at significantly higher levels and 2191 genes at significantly lower levels 3 h post inoculation in oestrus in comparison to pre-inoculation levels. In dioestrus, the expression of 1476 genes was up-regulated and 383 genes were down-regulated post inoculation. Many immune related genes were found to be up-regulated after the introduction of *E. coli*. These include pathogen recognition receptors, particularly toll-like receptors *TLR2* and *4* and NOD-like receptor NLRC5. In addition, several interleukins including *IL1B, IL6, IL8* and *IL1ra* were significantly up-regulated. Genes for chemokines, including *CCL 2, CXCL 6, 9, 10, 11* and *16* and those for antimicrobial peptides, including secretory phospholipase *sPLA*_*2*_, lipocalin 2, lysozyme and equine β-defensin 1, as well as the gene for tissue inhibitor for metalloproteinases *TIMP-1* were also up-regulated post inoculation.

**Conclusion:**

The results of this study emphasize the complexity of an effective uterine immune response during acute endometritis and the tight balance between pro- and anti-inflammatory factors required for efficient elimination of bacteria. It is one of the first high-throughput analyses of the uterine inflammatory response in any species and several new potential targets for treatment of inflammatory diseases of the equine uterus have been identified.

**Electronic supplementary material:**

The online version of this article (doi:10.1186/s12864-015-2139-3) contains supplementary material, which is available to authorized users.

## Background

Equine endometritis, a local inflammation of the superficial layers of the uterus, has been ranked as the third most common medical problem of adult horses by equine practitioners in the USA [1], with approximately 15 % of all Thoroughbred mares showing signs of persistent mating-induced endometritis (PMIE) [2]. The decrease in pregnancy rates in affected mares causes significant losses to horse breeding industries [3].

A transient endometritis is the physiological response to the deposition of spermatozoa, seminal plasma, cell debris and bacteria into the uterus during natural breeding or artificial insemination [4, 5] and has also been described in other mammals, including dogs [6].

Healthy mares are able to clear the contamination of their uterus within 6 h after breeding [7, 8]. In contrast, mares susceptible to PMIE show a prolonged inflammatory response lasting up to 96 h post breeding [7, 8], and bacterial uterine infections were found in 25-60 % of barren mares [9, 10]. The two most commonly isolated organisms, *Escherichia coli (E. coli)* and *Streptococcus equi subspecies zooepidemicus (Sc. zooepidemicus)* are both considered opportunistic pathogens [11], suggesting that the phenomenon may be more related to host factors than the virulence of the bacteria. Similar conditions can be seen in the genital tract of other species, including cattle [12], sows [13], bitches [14, 15] and humans [16], when increased numbers of bacteria meet a weak immune system.

Steroid hormone levels have a significant impact on the efficiency of the uterine immune response. In all species studied, the immune system faces greater challenges during oestrus, when the cervix is open, allowing vaginal bacteria and cell debris, sperm and bacteria introduced during breeding to enter the uterus. This is of particular importance in species in which the male deposits semen directly into the uterus, as is the case in horses, pigs and camelids [17]. Ovariectomised maiden mares treated with oestrogen begin clearing *Sc. zooepidemicus* within 2 h after inoculation, whereas mares treated with progesterone take 24 h to clear the same inoculum [18]. Similar observations have been made in ewes after inoculation with *Actinomyces pyogenes* and *E. coli* [19] and in gilts [20], rats [21] and bitches [22, 23] after inoculation with *E. coli*. In summary, bacterial growth, inflammatory cell migration and clinical illness seem to be correlated with high progesterone concentrations, while oestrus conditions significantly reduce the incidence of persistent infection and inflammation in the uterus.

Recent studies have shown that during oestrus the equine uterus develops a moderate to severe neutrophilia within 3 h after inoculation of *E. coli* [24] and by 8 h after insemination [25], with no accumulation of uterine fluid detected at any time after inoculation of the bacteria [24]. In contrast, the inoculation of *E. coli* into uteri in dioestrus led to purulent or serohaemorrhagic vaginal discharge starting 3 h after inoculation and signs of acute systemic inflammation by 6–12 h after inoculation. These changes were accompanied by recovery of heavy bacterial growths from endometrial samples taken 3 h after inoculation, whereas no bacteria could be cultured at the same time point in samples taken from uteri in oestrus [24, 26]. These findings suggest the equine uterus initiates a more efficient immune response during oestrus than in dioestrus.

At the molecular level, many of the toll-like receptors (TLR) expressed in mammals are present in the reproductive tract and exposure to gram-negative bacteria or LPS has been shown to enhance the expression of *TLR2* and *4* in mice [27], rabbits [28], dogs [29] and cattle [30].

Another important group of pattern recognition receptors (PRR) are the NOD-like receptors (NLR), which are responsible for the intracellular detection of pathogens. They can activate pro-inflammatory cytokines [31–33], but also regulate the immune response [34, 35].

Downstream of these receptors, the pro-inflammatory cytokines interleukin (IL) *IL1B*, *IL6* and *IL8*, as well as tumour necrosis factor α (TNFA) were shown to peak at 3 h after inoculation with *E.* coli regardless of cycle stage. In contrast, expression of mRNA for serum amyloid A and the anti-inflammatory interleukin 10 peaks 3 h after bacterial inoculation in dioestrus, with no significant change seen in oestrus [24, 36]. Similarly, cytokine expression is significantly altered during endometritis in bovine [37] and human uteri [38].

Additionally, mucous membranes, including the endometrium, have a range of antimicrobial molecules in their arsenal. These include factors that destabilize bacterial cell walls, such as defensins, lysozyme and secreted phospholipase A_2_ (sPLA_2_) [39, 40], and those that inhibit bacterial enzymes, such as secretory leukoprotease inhibitor (SLPI), which is also known as equine neutrophil antimicrobial peptide 2 (eNAP-2) [41–43]. Bacteriostasis can be achieved by binding elements essential for the microbial metabolism such as iron, as is described for lipocalin 2, also known as uterocalin [44] and lactoferrin [40, 45]. Uteroferrin, also known as tartrate-resistant acid phosphatase (ACP 5 or TRAP) has been shown to be essential in trans-placental transport of iron in pigs and horses [46, 47].

Finally, matrix metallopeptidases (MMP) have been implicated in the activation and regulation of several participants in the immune response [48–50]. In the equine uterus, MMP2 and 9 have been shown to be significantly up-regulated 5 h after inoculation with *Sc. zooepidemicus* in both oestrus and dioestrus [51].

No study has directly compared the effect ovarian hormone concentrations have on global gene expression in response to the introduction of bacteria in the equine uterus. Using high-throughput sequencing, significant alterations in gene expression have been detected in dogs with pyometra in comparison to healthy control animals. The genes with the most highly up-regulated levels of expression in this study were associated with inflammatory processes, such as cytokine production and inflammatory cell extravasation, as well as with processes such as proteolysis [52]. Our previous studies have detected significantly different mRNA expression profiles between oestrus and dioestrus in horses, with several pathways related to the immune response containing more genes expressed at significantly higher levels in oestrus than in dioestrus [53]. While the levels of expression of uterine immune response genes tend to be up-regulated during oestrus in mice [54], in bovine and human uteri a greater number of immune genes are expressed at higher levels during dioestrus [55–57].

Limited information is available detailing the initial hours of uterine immune response to pathogens, even though this is the most significant period for rapid physiological clearance of foreign material. Our knowledge of the influence of ovarian hormones on this initial uterine immune response is even more restricted. The objective of our study was to characterize alterations in patterns of gene expression in the equine endometrium in response to the introduction of bacterial pathogens. In addition, we explored the effect that steroid hormone concentrations associated with different oestrus cycle stages have on these expression patterns.

## Materials and methods

### Selection of experimental animals

Endometrial biopsies were obtained from five Standardbred mares. All horses were aged between 3–4 years and were maintained on pasture at the facilities of the Faculty of Veterinary and Agricultural Sciences, The University of Melbourne, Australia. Treatments and procedures were approved by the University of Melbourne Animal Ethics Committee (Approval Number: 1112297).

The mares used in this study were clinically healthy and were confirmed to be resistant to post-breeding endometritis (PMIE) by insemination with frozen stallion semen to assess whether this induced uterine inflammation, a reliable indicator of susceptibility to PMIE in mares [59]. None of the mares developed clinical signs of persistent inflammatory oedema 24 h after insemination or histopathological changes in endometrial tissues. Conventional bacteriological culture of uterine swabs was used to confirm the absence of any pre-existing bacterial infection of the uterus of each mare.

### Preparation of E. coli inocula

An *E. coli* strain isolated from a mare susceptible to post-breeding endometritis and stored at −80 °C was streaked onto a Mueller-Hinton agar plate and incubated for 24 h (h) at 37 °C. A single colony was transferred to 2 ml Mueller-Hinton broth and the culture was incubated overnight at 37 °C. The overnight broth culture was diluted to achieve a final concentration of 10^5^ colony forming units (CFU) per inoculum. The bacteria were kept on ice for a maximum of 1 h before use.

### Inoculation of E. coli and collection of endometrial tissue and swap samples

Follicular development, intrauterine fluid, development of uterine oedema and cervical and uterine tone were monitored daily by trans-rectal palpation and ultrasonographic examination. In addition, rectal temperature, heart rate and respiratory rate were monitored daily.

Uterine oedema was evaluated using a five tier oedema score system (E0-E4) with E0 representing the absence of any uterine oedema and E4 representing pathological inflammatory oedema. Similar four to six tier scores have been described previously [60–62].

Mares were inoculated with *E. coli* in two consecutive oestrous cycles in a crossover study design. Three of the mares were randomly assigned to group 1 and inoculated with *E. coli* during oestrus (based on the presence of a dominant follicle ≥ 35 mm in diameter, uterine oedema and decreased uterine and cervical tone). Endometrial samples were obtained by trans-cervical biopsy before (0 h) and 3 h after inoculation using an alligator jaw biopsy punch (Jorvet, Loveland, CO, USA) and a sample for uterine culture was collected using a double-guarded swab (Minitube Australia, Ballarat, Vic, Australia). Care was taken to take biopsies from different sites near the base of a uterine horn at each time point.

After establishing the absence of inflammation at the following oestrus, mares were inoculated with the same strain of *E. coli* during dioestrus, as determined by detection of a functional corpus luteum and the absence of uterine oedema five days after ovulation. Plasma progesterone concentrations were evaluated from samples collected prior to inoculation. Endometrial biopsies and swabs were again taken before (0 h) and 3 h after inoculation.

The two mares assigned to the second group were subjected to the same procedures in reverse order, being initially inoculated during dioestrus and subsequently during oestrus. Samples were taken at the same time points using the same methods as described above (Additional file 1: Figure S1).

All endometrial biopsies were obtained from the base of the uterine horns and cut with a sterile scalpel blade directly after collection to allow for one part to be used for microbiology, while the other was processed for next-generation sequencing.

### Preparation of samples for microbiology

The portion of the biopsy to be used for microbiology and the uterine swab sample were plated onto a Mueller-Hinton-Agar plate and incubated aerobically at 37 °C for 24 h to identify and quantify the bacteria in the uterus. Colony counts were scored as: no growth, <5 CFU; mild growth, 5–10 CFU; moderate growth, 11–50 CFU; and heavy growth, > 50 CFU, as described previously [26].

Representative colonies from each plate were replated onto MacConkey agar to assess their capacity to ferment lactose and were also tested for their capacity to produce indole from tryptophan to confirm their identity as *E.coli*.

### Preparation of endometrial samples for sequencing

The second part of the endometrial biopsy was immediately placed in RNAlater (Life Technologies Australia, Mulgrave, Vic, Australia), incubated overnight on a rocking platform at room temperature, then stored at −80 °C until further processed. These samples were homogenized in Trizol (Qiagen, Chadstone, Vic, Australia) using a Polytron homogeniser (IKA Works, Selangor, Malaysia) and total RNA was purified using the RNeasy Universal Plus Mini Kit (Qiagen) according to the manufacturer’s instructions. Each total RNA pellet was resuspended in 70 μl RNAse-free water and the nucleotide concentration and purity was assessed for each sample by spectrophotometry using a NanoDrop ND-1000 (Thermo Fisher Scientific Australia Pty Ltd, Scoresby, Vic, Australia). All samples had A_260_/A_280_ ratios greater than 1.99. RNA integrity was verified by visualizing distinct 28S and 18S ribosomal RNA bands in 1 μl of total RNA electrophoresed through 1 % agarose.

A 12–14 μg sample of RNA from each biopsy was dried in RNAstable tubes (Biomatrica, Belrose, Vic, Australia) according to the manufacturer’s instructions and shipped at room temperature to the Institute of Biotechnology, Cornell University, Ithaca, NY, USA. After transport, RNA integrity was verified using a 2100 BioAnalyzer (Agilent, Santa Clara, CA, USA).

### mRNA library preparation and next generation sequencing

Total RNA from each sample was reverse transcribed into a cDNA sequencing library using the Illumina TruSeq RNA Sample Preparation kit according to the manufacturer’s instructions (Illumina, San Diego, CA, USA). Products were paired-end sequenced using Illumina HiSeq sequencing at the Institute of Biotechnology, Cornell University, Ithaca, NY, USA. Two technical replicate sequencing runs were performed – one generating 55 base pair (bp) and the second 100 bp paired-end nucleotide reads.

### Data processing

The raw reads were filtered to remove low-quality sequences (PHRED score of ≤ 20), short reads (<30 nucleotides in run 1 and <40 nucleotides in run 2) and sequencing adapters using Trimmomatic software [63]. FastQC version 0.10.0 was used to assess the quality of the filtered reads [http://www.bioinformatics.babraham.ac.uk/projects/fastqc/].

High quality paired reads (96.7-97.8 % of the raw data) were mapped to the annotated equine genome (EquCab_2.71) [64] using bowtie2 [65] and gene transcription was inferred using RSEM version 1.2.11 [66].

Initially, logarithmically normalized exonic read density values of the technical replicates sequenced from the oestrus and dioestrus cDNA libraries from uteri with and without bacteria were shown to be consistent by regression analysis performed in R (version 2.15.2). All further analyses were performed with merged technical replicates.

### Gene expression analysis

After the removal of genes with low read counts (only keeping genes with > 1 read/ million in > 3 samples) and normalization for sequencing library size (using the trimmed mean of M-values [67]), differences in gene transcription (adjusted *P* < 0.05 [68] and a log_2_ fold change (logFC) > |1|) were determined using edgeR version 3.0.8 [69]. The BioMart annotation tool [64] was used to extract annotations for all differentially transcribed genes. For additional stringency, genes with a deviation of less than 40 reads per million between pre- and post-inoculation levels were filtered out to create an additional gene list. When this filter was used, it is stated in the results section.

A cluster analysis was performed using the R package gplots to determine whether the treatment order changed the overall gene expression profiles of a sample.

### Enrichment of genes within gene ontology categories and associated biological pathways

The Bioconductor R package GOSeq v1.10.0 [70] was used in combination with the annotation package GO.db v2.8.0 [71] to further analyse differentially expressed genes. The GOSeq default method “Wallenius” was used after calculating the Probability Weighting Function (PWF) to identify significantly enriched gene ontology (GO) categories (adjusted *P* < 0.05) when analysing genes up-regulated after bacterial inoculation in either oestrus or dioestrus. The GO TERM information provided in the GO.db package was then used to annotate significantly enriched categories with their name and their ontology (biological process (GO:BP), molecular function (GO:MF) or cellular component (GO:CC)). The number of up-regulated genes was calculated for each enriched category.

The Kyoto encyclopaedia of genes and genomes (KEGG) database [72] was used to conduct a pathway enrichment analysis to identify pathways containing more up-regulated genes than expected by chance after inoculation of bacteria. To increase the clarity of the results, pathways associated with the first level category “Human Diseases” were removed from the analysis.

Images were produced that depict differentially expressed genes within enriched pathways using the advanced KEGG Pathway mapping tool “Search & Color Pathway” in the KEGG Pathway database (http://www.genome.jp/kegg/tool/map_pathway2.html).

## Results

### Clinical and gynaecological examination

No significant changes were detected in rectal temperature, heart rate or respiratory rate before and three hours after bacterial inoculation.

All mares identified as being in the dioestrus phase of the cycle were found to have plasma progesterone concentrations of between 25 and 33 nmol/L, as expected.

There was no intrauterine fluid accumulation at the time of inoculation in any of the mares. Oedema scores prior to inoculation of *E. coli* ranged from E2 to E3 during oestrus, while no oedema could be detected in any of the dioestrus uteri (E0). Three hours after inoculation with *E. coli*, all mares had significant inflammatory oedema (E4) detectable by ultrasonographic examination in both cycle stages. Slight fluid accumulation was detected three hours after inoculation in two mares during oestrus and in one mare during dioestrus.

### Microbiology

No bacteria were cultured from any of the endometrial biopsies or uterine swabs collected prior to inoculation with *E. coli.* Three hours after inoculation, no to moderate bacterial growth was detected in endometrial biopsy samples taken in oestrus (0–20 CFU), while the uterine swabs yielded mild to heavy growth (3 to >50 CFU). In dioestrus, inoculation of *E. coli* resulted in moderate growth in one biopsy sample (32 CFU) and heavy growth in all other biopsy and swab samples. All colonies sampled were identified as *E. coli*.

### Read alignment and differential gene expression

The total number of paired reads per sample after quality filtering ranged from 3.31 to 19.96 million reads/sample with an average of 10.51 million reads/sample.

A significant correlation was detected between the logarithmically normalized exonic read density values for each technical replicate (0.9574 ≤ R^2^ ≤ 0.9638), so sequencing data from technical replicates was merged for each of the biological replicates at each time point, i.e. pre- and post-inoculation in oestrus and dioestrus, respectively.

The cluster analysis revealed that the treatment order did not have an impact on the gene expression profiles, with samples not clustering according to treatment group, but 19 out of the 20 samples clustering by inoculation status and cycle stage, with one post-inoculation dioestrus sample clustering with the pre-inoculation samples (Additional file 2: Figure S2).

Significant differences in gene expression have been detected between the two cycle stages prior to bacterial inoculation, as described previously [73]. With the stricter criteria used in this study to define significantly differentially expressed genes (log_2_ fold change > |1| rather than > |0.5|), 1187 and 1188 genes were still found to be transcribed at higher levels in oestrus and in dioestrus, respectively.

**Fig. 1:**
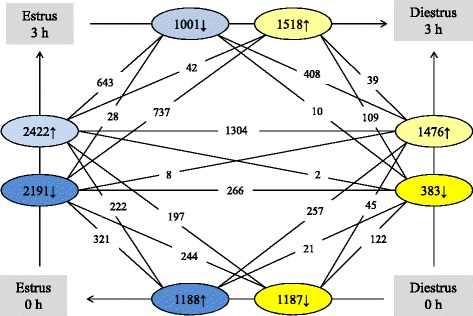
Graph of all differentially expressed gene counts. Numbers in ellipsoid structures represent genes up- or down-regulated at the time point towards which the arrow they are sitting on is pointing in comparison to the time point from which the arrow is starting. All other numbers represent genes found in the intersecting set of both ellipsoid structures to which they connect

A large number of genes were found to be differentially expressed 3 h after the inoculation with bacteria in both oestrus and dioestrus. To identify the impact of the hormone environment on the immune response to bacteria, genes expressed differentially pre- and post-inoculation were compared between cycle stages and to genes already differentially expressed prior to inoculation. Additionally, post-inoculation levels were compared between the cycle stages and the intersecting set of genes found differentially expressed both between the 3 and 0 h samples and between the two 3 h sample groups were identified (Fig. 1).

Overall, the expression of 2422 genes was found to be up-regulated after bacterial inoculation in oestrus compared to pre-inoculation levels, while 2191 genes were down-regulated following the introduction of bacteria. In dioestrus, the inoculation of bacteria resulted in significantly higher levels of expression of 1476 genes and significantly lower levels of expression of 383 genes (Fig. 1).

The expression of 1304 genes was found to be up-regulated following inoculation of *E. coli* in both oestrus and dioestrus (Fig. 1). The genes with the greatest fold change between pre- and post-inoculation levels within this group included those for several interleukins, chemokines, matrix metallopeptidases and antimicrobial peptides.

The expression of 840 genes was found to be up-regulated after inoculation with *E. coli* in oestrus but constitutively transcribed or down-regulated after inoculation with *E. coli* during dioestrus. This group contained the genes for beta defensins 2 and 3, matrix metallopeptidase 7 and interleukin 36α. However, additional filtering of genes that had a deviation of less than 40 reads per million between pre- and post-inoculation levels, left only 173 genes in this group. These included those for cathepsin L1, retinol-binding protein 4 precursor, lactotransferrin, the antimicrobial peptide secretory phospholipase A2 and 11 different solute carrier families.

Only 88 genes were up-regulated during dioestrus following the bacterial inoculation, but constitutively transcribed or down-regulated during oestrus. The genes for the chemokine receptor CXCR4 and cathepsin E were amongst the 27 genes that remained after filtering out those that had deviations of less than 40 reads per million between pre- and post-inoculation levels.

The expression of 266 genes, including that for interferon ε, was significantly lower after the introduction of bacteria in oestrus but not dioestrus, while 1689 genes were only expressed at significantly reduced levels during oestrus, including those for the chemokine ligand CCL28 and receptors CX3CR1 and CXCR4 and tumour necrosis factor receptor 11a. In contrast, 62 genes were expressed at significantly lower levels in dioestrus, but not oestrus, after inoculation of *E. coli*.

A total of 643 genes were found to be expressed at significantly higher levels in oestrus 3 h post inoculation compared to both oestrus pre-inoculation levels and also dioestrus post-inoculation levels. These genes included those for MMP7, the glycoprotein olfactomedin 4, which is associated with several inflammatory conditions, the interleukins IL 11 and IL36α, the antimicrobial peptides beta defensin 2, and beta defensin 3 and lacto(trans)ferrin, the granulocyte colony-stimulating factor CSF3, the chemokine CXCL6, the chemokine receptor CXCR7 and members of the tumour necrosis factor receptor superfamily CD40 and TNFRSF9.

Of the 2191 genes that were down-regulated in oestrus after the inoculation of bacteria, 737 were expressed at lower levels 3 h post-inoculation in oestrus compared to their expression 3 h post-inoculation in dioestrus. These genes included those for interleukin 17 receptor E, interferon ε and aquaporin 5. Only 39 genes were up-regulated after the introduction of bacteria in dioestrus in comparison to pre-inoculation levels and expressed at higher levels in dioestrus after inoculation with *E. coli* compared to oestrus after inoculation with *E. coli*. These included the genes for serum amyloid A, which was expressed at 4 times higher levels in dioestrus 3 h after inoculation with *E. coli* than in oestrus 3 h at the same time point. Only 10 genes were down-regulated in dioestrus after inoculation with *E. coli* and expressed at lower levels in dioestrus than in oestrus after inoculation with *E. coli*. Fig. 1 summarizes these data.

### Gene ontology and pathway analyses

The majority of genes up-regulated after inoculation with *E. coli* could be placed in immune related GO categories and KEGG pathways.

In both cycle stages, all genes in multiple immune-related BP categories were up-regulated after inoculation with *E. coli,* including those for positive regulation of CD4-positive α/β T-cell differentiation (4/4 genes), of immune response (3/3 genes) and of lymphocyte differentiation (4/4 genes). Similarly, all genes in categories for mast cell activation (4/4 genes), neutrophil activation (3/3 genes) and regulation of phagocytosis (4/4 genes) were also up-regulated after inoculation with *E. coli*. In the innate immune response category, 69 (25 /36 genes) and 56 % (20/ 36) of genes were up-regulated after inoculation with *E. coli* in oestrus and dioestrus, respectively. The most significantly enriched BP categories are listed in Table 1.

**Table 1:** Most enriched gene ontology biological processes after bacterial inoculation sorted by *P*-value

Category	Up-regulated after bacterial infection^a^		Down-regulated after bacterial infection^b^	
	Oestrus	Dioestrus	Oestrus	Dioestrus
Immune system process	310 (40 %)	241 (31 %)	51 (7 %)	9 (1 %)
Immune response	113 (52 %)	89 (41 %)	5 (2 %)	1 (0 %)
Inflammatory response	50 (57 %)	49 (56 %)	2 (2 %)	0 (0 %)
Response to stress	295 (25 %)	216 (18 %)	132 (11 %)	19 (2 %)
Cytokine-mediated signaling pathway	34 (54 %)	31 (49 %)	4 (6 %)	0 (0 %)
Response to lipopolysaccharide	36 (58 %)	30 (48 %)	4 (6 %)	0 (0 %)
Defense response to virus	28 (54 %)	27 (52 %)	1 (2 %)	0 (0 %)
Innate immune response	25 (69 %)	20 (56 %)	1 (3 %)	0 (0 %)
Positive regulation of B cell proliferation	20 (67 %)	17 (57 %)	0 (0 %)	0 (0 %)
Antigen processing and presentation	41 (55 %)	25 (34 %)	0 (0 %)	0 (0 %)
Positive regulation of I-kappaB kinase/NF-kappaB signaling	48 (42 %)	32 (28 %)	9 (8 %)	0 (0 %)
Antigen processing and presentation of peptide antigen via MHC class I	17 (81 %)	6 (29 %)	0 (0 %)	0 (0 %)

^a^The number of genes up-regulated after bacterial infection in each category in oestrus and dioestrus and what percentage that represents of the total number of genes in this category

^b^The number of genes down-regulated after bacterial infection in each category in oestrus and dioestrus and what percentage that represents of the total number of genes in this category

Categories assigned to molecular functions (GO:MF) confirmed this immunity-related bias. The most significantly enriched categories in this group were those for cytokine activity, chemokine activity, cytokine receptor activity and tumour necrosis factor receptor binding in both cycle stages.

Accordingly, most products of genes up-regulated after inoculation with *E. coli* were found in the plasma membrane or outside the cell (extracellular space), as would be expected for the paracrine and endocrine signalling molecules of the innate immune system. In addition, 81 (13/16) and 75 % (12/16) of the genes in the GO:CC category MHC class II protein complex were up-regulated in oestrus and dioestrus, respectively, while the MHC class I protein complex contained 23 (53 %) genes up-regulated in oestrus and only 8 (19 %) up-regulated in dioestrus.

In the KEGG pathways, 12 out of 16 immune pathways were enriched after inoculation with *E. coli* in both cycle stages (Table 2). In addition, several immune-related signal transduction pathways, including the MAPK, NFκB and TNF signalling pathways and the pathway for cytokine-cytokine receptor interaction, were also enriched after inoculation with *E. coli* in both oestrus and dioestrus (Table 2). The 5 pathways most significantly enriched 3 h after inoculation with *E. coli* in oestrus were those for osteoclast differentiation, natural killer cell mediated cytotoxicity, TNF signalling, cytokine-cytokine receptor interaction and chemokine signalling. The phagosome (position 6 in oestrus) and Jak-STAT signalling pathways (position 13 in oestrus) were also enriched in dioestrus, while the natural killer cell mediated cytotoxicity (position 8 in dioestrus) and the chemokine signalling pathways (position 6 in dioestrus) were slightly lower on a list sorted by *P* value.

**Table 2:** Enriched KEGG pathways in the category immune system (blue) and immune-related signal transduction molecules (green) after bacterial inoculation

KEGG pathway	Up-regulated genes after inoculation with *E. coli* ^a^		Comparison of gene counts 3 h after inoculation with *E. coli* ^b^		Genes with higher expression in oestrus at 3 h after inoculation compared to dioestrus at 3 h after inoculation^c^		Total number of genes in pathway
	Oestrus	Dioestrus	Higher in oestrus	Higher in dioestrus	Up-regulated in oestrus after inoculation	Up-regulated in dioestrus after inoculation	
Antigen processing and presentation	73* (32)	52* (25)	15	3	5	4	219
B cell receptor signaling pathway	52* (12)	35* (12)	13	4	5	4	112
Chemokine signaling pathway	107* (5)	73* (6)	27*	13	13	10	190
Complement and coagulation cascades	32	29* (29)	19*	12	13*	12*	81
Cytosolic DNA-sensing pathway	34* (27)	24* (21)	10	0	5	0	95
Fc epsilon RI signaling pathway	48* (17)	30* (18)	13	6	6	5*	184
Fc gamma R-mediated phagocytosis	64* (11)	36* (20)	16	11	4	4	71
Hematopoietic cell lineage	54* (16)	46* (9)	16*	5	12*	10*	183
Intestinal immune network for IgA production	39	31* (39)	5	0	3	3	227
Leukocyte transendothelial migration	78* (19)	50* (16)	32*	12	12	9	118
Natural killer cell mediated cytotoxicity	100* (2)	65* (8)	28*	4	13*	9	244
NOD-like receptor signaling pathway	41* (18)	30* (14)	11	5	8*	7*	129
RIG-I-like receptor signaling pathway	35* (30)	27* (19)	10	6	6	5	114
T cell receptor signaling pathway	66	36	16	7	8	5	96
Toll-like receptor signaling pathway	65* (8)	50* (7)	19*	6	11*	9*	78
Cell adhesion molecules (CAMs)	93* (43)	75* (15)	29	13	11	8	320
Cytokine-cytokine receptor interaction	129* (4)	109* (2)	52*	14	36*	32*	273
Jak-STAT signaling pathway	80* (13)	65* (5)	33*	6	23*	21*	181
MAPK signaling pathway	120* (9)	78* (11)	48*	21	26*	18*	285
NF-kappa B signaling pathway	76* (35)	55* (23)	18	5	8	7	241
TNF signaling pathway	79* (3)	69* (3)	28*	9	19*	17*	131

^*^Significantly enriched KEGG pathway (P-value < 0.05)

^a^The number of genes significantly up-regulated after bacterial inoculation in each pathway (and the rank of this pathway in comparison to all pathways found in oestrus or dioestrus sorted by P value)

^b^The number of genes that were found to be expressed at significantly higher levels in one cycle stage 3 h after inoculation in comparison to the other cycle stage 3 h after inoculation

^c^The number of genes that were found to be expressed at significantly higher levels in oestrus 3 h after inoculation in comparison to dioestrus 3 h after inoculation and were also found to be significantly up-regulated after inoculation when compared to pre-inoculation levels in oestrus or dioestrus

Genes expressed at lower levels after the inoculation of bacteria were mostly found in metabolism pathways and the genetic information processing subcategory replication and repair.

There was a clear difference between the pathways enriched among genes differentially expressed in oestrus 3 h post-inoculation in comparison to genes differentially expressed in dioestrus at the same time point. While the most enriched pathways in oestrus were associated with the ECM receptor interaction, cytokine-cytokine receptor, focal adhesion, PI3K-Akt-signalling and MAPK signalling pathways, most genes expressed at higher levels in dioestrus were in the amino acid, lipid and carbohydrate metabolism and peroxisome pathways.

Finally, of the intersecting set of genes that were up-regulated 3 h post inoculation in oestrus compared to pre - inoculation levels, and also expressed at significantly higher levels in oestrus in comparison to dioestrus 3 h post inoculation, the cytokine-cytokine receptor interaction pathway was the one most significantly enriched, followed by the Jak-STAT and TNF signalling pathways. The same pathways were enriched among the genes that were up-regulated in response to *E. coli* in dioestrus, but still expressed at higher levels in oestrus samples 3 h post-inoculation with *E. coli* in comparison to dioestrus 3 h post-inoculation with *E. coli*. In contrast, the genes more highly expressed in dioestrus than in oestrus 3 h after inoculation with *E. coli* and also more highly expressed post-inoculation than prior to inoculation in dioestrus did not cluster in any specific pathways.

### Spotlight on innate immunity

As the main focus of this study was the initial uterine immune response to the introduction of *E. coli*, genes associated with innate immunity were analysed in greater depth.

#### Pathogen recognition receptors

**Fig. 2:**
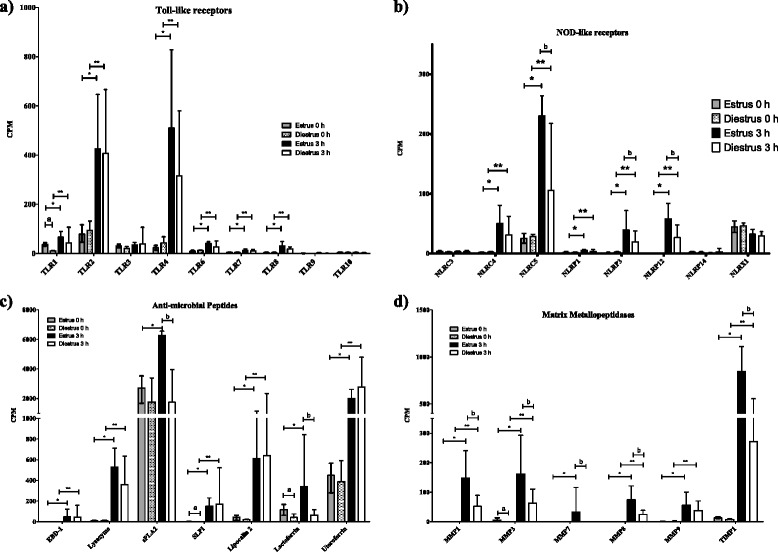
mRNA transcript levels of endometrial genes of TLR, NLR, antimicrobial peptides and MMP. The mRNA transcript levels of genes associated with **a** toll-like receptors (TLR), **b** NOD-like receptors (NLR), **c** antimicrobial peptides and **d** matrix metallopeptidases (MMP). Gene expression is presented in copies per million reads (CPM), bars represent the mean CPM values, error bars the range. Significant differences (adjusted *P* < 0.05) between post and pre inoculation expression are indicated by asterisks: ‘*’ represents significant differences between estrus samples and ‘**’ significant differences between dioestrus samples. Letters indicate significant differences between cycle stages: ‘a’ represents a significant difference between oestrus and dioestrus before inoculation, and ‘b’ between oestrus and dioestrus after inoculation

**Fig. 3:**
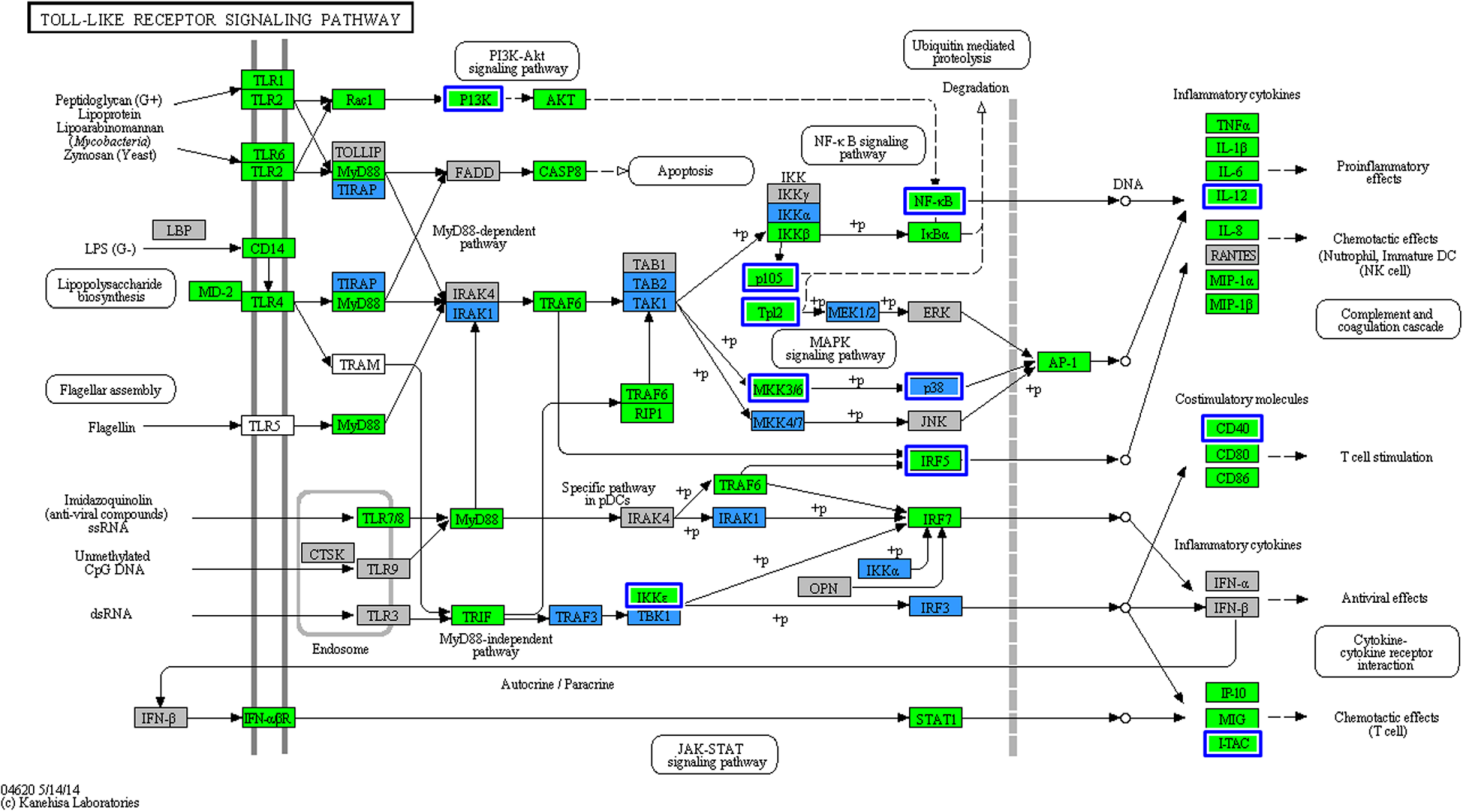
Differentially expressed genes in the toll-like receptor signalling pathway (ko04620). Genes significantly up-regulated after inoculation of bacteria in both cycle stages are highlighted in green, genes only significantly up-regulated after inoculation in oestrus are highlighted in blue and blue boxes around genes represent genes that were significantly higher in oestrus 3 h post-inoculation in comparison to dioestrus 3 h post inoculation. Genes in grey boxes were present in the horse transcriptome, but not differentially expressed, while genes in white boxes were not described as horse KO terms. The figure was created using the KEGG “Search and Color Pathway” tool

Expression of genes of both the NLR and the TLR family were up-regulated after the inoculation of *E. coli* (Figs. 2a, b and 3). These changes are also apparent in the KEGG pathways, in which 83 (65/78) and 64 % (50/78) of genes in the TLR pathway were expressed at significantly higher levels after inoculation with bacteria in oestrus and dioestrus, respectively. In the TLR family, the biggest changes were detected in *TLR2* and *4* mRNAs with logFCs of 2.7 and 4.7 in oestrus and 2.2 and 3.0 in dioestrus, respectively. In addition, the gene expression of TLR4 helper molecules CD 14 and MD 2 was similarly up-regulated, with logFCs of 3.8 and 2 in oestrus and 3.3 and 1.1 in dioestrus, respectively, while those for the lipopolysaccharide-binding protein (LBP) were only detected at minimal levels in all samples. In addition, many genes for proteins that lie in pathways downstream of these receptors were also expressed at significantly increased levels (Fig. 3).

The NLR signalling pathway was also significantly enriched in both oestrus and dioestrus after inoculation with *E. coli* (Table 2). There was a significant increase in the copy numbers of NLRC4 and 5 mRNAs, with log_2_ fold changes (logFC) of 4.9 and 3.5 in oestrus and 3.6 and 1.8 in dioestrus, respectively. The abundance of *NLRP3* and *12* mRNAs increased by logFCs of 4.9 and 8.9 in oestrus and 3.0 and 6.8 in dioestrus, respectively.

#### Cytokines

**Fig. 4:**
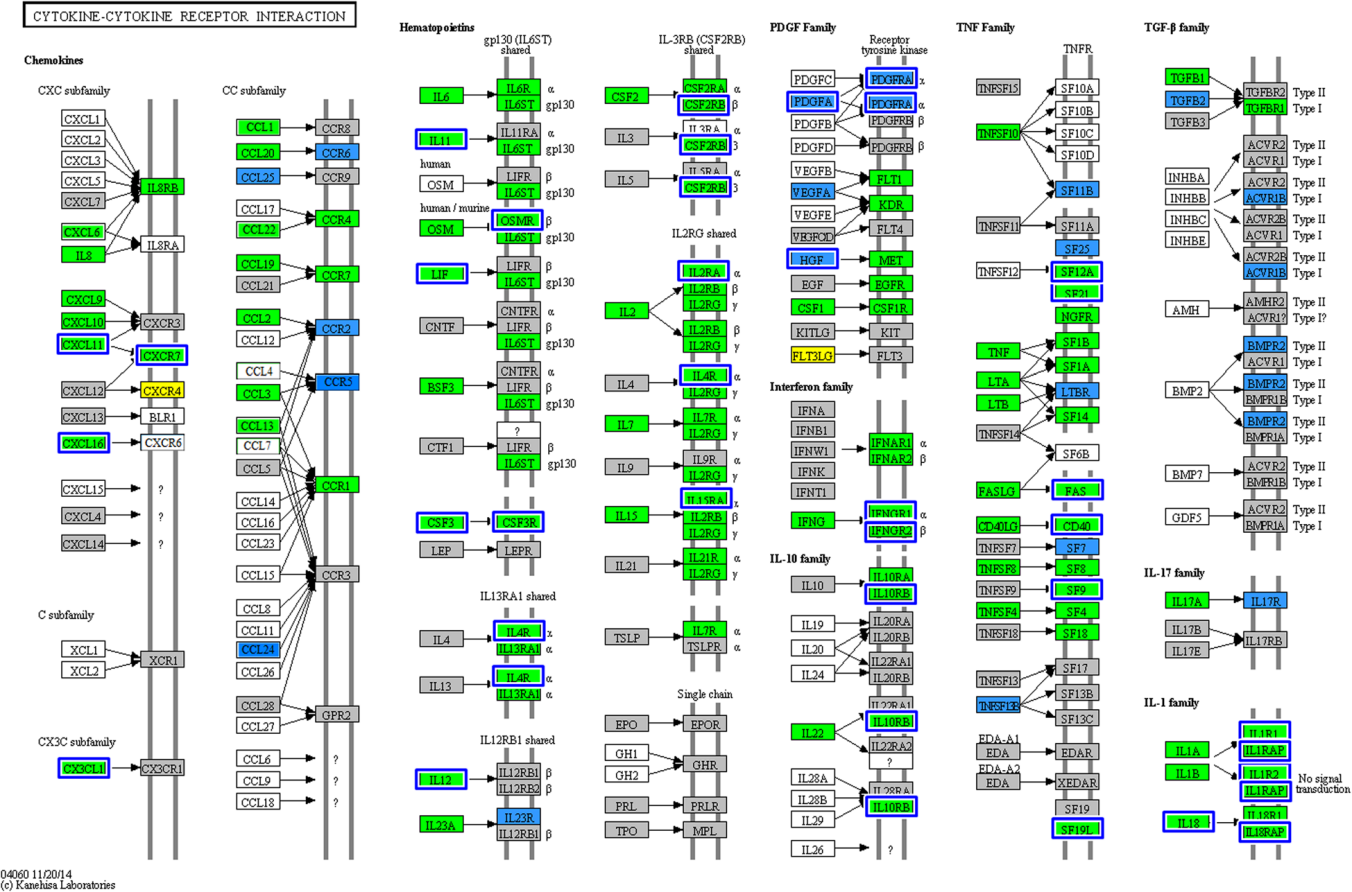
Differentially expressed genes in the cytokine-cytokine receptor interaction pathway (ko04060). Genes significantly up-regulated after inoculation of bacteria in both cycle stages are highlighted in green, genes only significantly up-regulated after inoculation in oestrus are highlighted in blue, those only significantly up-regulated in dioestrus in yellow and blue boxes around genes represent genes that were significantly higher in oestrus 3 h post-inoculation in comparison to dioestrus 3 h post inoculation. Genes in grey boxes were present in the horse transcriptome, but not differentially expressed, while genes in white boxes were not described as horse KO terms. The figure was created using the KEGG “Search and Color Pathway” tool

**Fig. 5:**
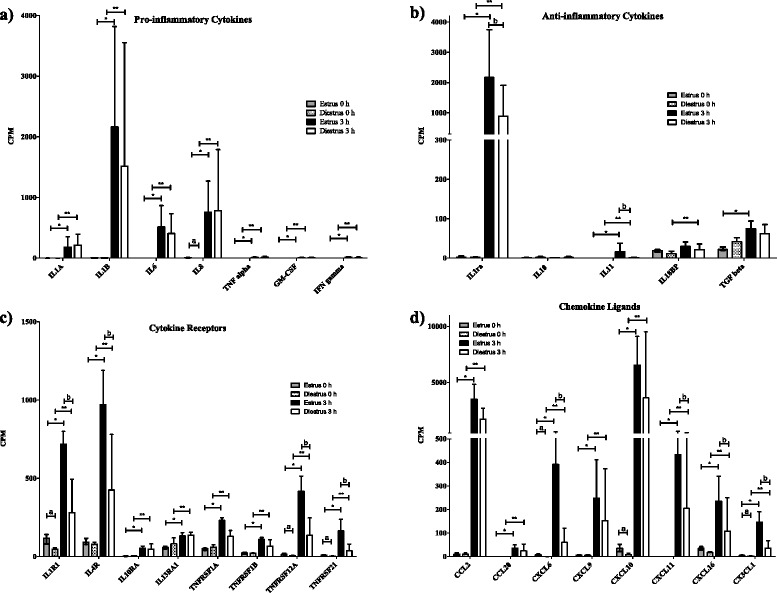
mRNA transcript levels of endometrial genes associated with cytokines, cytokine receptors and chemokines. The mRNA transcript levels of endometrial genes associated with **a** pro-inflammatory cytokines, **b** anti-inflammatory cytokines, **c** cytokine receptors and **d** chemokine ligands. Gene expression is presented in copies per million reads (CPM), bars represent the mean CPM values, error bars the range. Significant differences (adjusted *P* < 0.05) between post- and pre - inoculation expression are indicated by asterisks: ‘*’ represents significant differences between oestrus samples and ‘**’ significant differences between dioestrus samples. Letters indicate significant differences between cycle stages: ‘**a**’ represents a significant difference between oestrus and dioestrus before inoculation, and ‘**b**’ between oestrus and dioestrus after inoculation

Cytokine-mediated signalling pathways were significantly enriched in both oestrus and dioestrus following infection with *E. coli*. Significant enrichment was also detected when comparing oestrus samples after inoculation to dioestrus samples after inoculation (Tables 1 and 2). Genes for several pro- and anti-inflammatory cytokines were significantly up-regulated 3 h post-inoculation in comparison to pre-inoculation levels (Figs. 4 and 5a, b). The most noticeable increase in expression of genes for pro-inflammatory cytokines was seen for the *IL1B* precursor, for which average normalized counts across samples from the 5 mares increased from 4 to 2164 and 1516 copies per million reads (CPM) in oestrus (logFC of 10) and dioestrus (logFC of 8.5), respectively. A smaller, but still very apparent, increase was seen in the expression of genes for *IL6* (logFCs of 9.8 in oestrus and 11.1 in dioestrus), the *IL8* precursor (logFCs of 7.5 in oestrus and 11.5 in dioestrus) and *IL1α* (logFCs of 8.2 in oestrus and 9.2 in dioestrus) (Fig. 5a). Levels of expression of genes for other pro-inflammatory cytokines, including those for TNF, granulocyte-macrophage colony stimulating factor (GM-CSF) and interferon-γ (IFNG), showed only relatively small increases (CPM values), from no expression before exposure to *E. coli* to levels of below 100 after exposure (Fig. 5a). The caspase-1 gene showed only a slight increase after inoculation with *E. coli,* from 4.0 to 20.2 CPM in oestrus and 2.8 to 15.7 CPM in dioestrus.

Among genes for anti-inflammatory cytokines, the expression of IL1 receptor antagonist (*IL1ra*) showed by far the greatest up-regulation post-inoculation, with logFCs of 10 (from 2.5 up to 2171 CPM) and 8 (from 2.6 to 889 CPM) in oestrus and dioestrus, respectively. This was reflected in significantly higher levels of expression in oestrus after inoculation than in dioestrus after inoculation (logFC of 1.97). The genes for all other anti-inflammatory cytokines, including those for IL4, IL10, IL11 and IL13, and those for the IL18 binding protein and transforming growth factor-β (TGFB), were detected at an average level of below 100 CPM (Fig. 5b).

Among the genes for cytokine receptors, those for the receptors for IL1, IL4, IL10 and IL13 were expressed at significantly higher levels post-inoculation than pre-inoculation in both oestrus and dioestrus. In addition, the genes for the IL1 receptor 1 and the IL4 receptor were expressed at significantly lower levels post-inoculation in dioestrus than in oestrus post-inoculation. Genes for receptors for the TNF receptor superfamily (TNFRSF) were also significantly up-regulated in response to *E. coli* in oestrus and to a lesser extent in dioestrus (Fig. 5c).

#### Chemokines

The chemokine signalling pathway was significantly enriched by KEGG pathway analysis for both oestrus and dioestrus after inoculation with *E. coli*. Significant enrichment of the chemokine signalling pathways was seen at oestrus 3 h post-inoculation, in comparison to dioestrus 3 h post-inoculation (Table 2).

In the CCL family, the gene for CCL2 had the highest fold-change in transcription after inoculation with *E. coli*, with logFCs of 8.7 and 7.4 in oestrus and dioestrus, respectively (Fig. 5d). This corresponded to average CPM values of 10 before and 3523 (oestrus) and 1745 (dioestrus) after inoculation. Expression of the gene for CCL20 was not detected prior to inoculation with *E. coli*, but increased to levels of 35 CPM in oestrus and 24 CPM in dioestrus following exposure to *E. coli*.

The most strongly expressed chemokine gene in the CXCL family after inoculation of *E. coli* was that for CXCL10 (Fig. 5d), the expression of which increased from an average of 35 in oestrus and 9 CPM in dioestrus before inoculation to 6553 in oestrus and 3930 in dioestrus after inoculation of *E. coli*. While CPM values in oestrus and dioestrus samples taken prior to inoculation were relatively similar within the groups, there was a large inter-sample variation after inoculation, with values ranging from 3843 to 9111 CPM in oestrus and from 382 to 9523 CPM in dioestrus. Other significantly differentially expressed members of this family include the genes for CXCL6, which increased from an average of 8 to 391 in oestrus (logFC of 5.8) and 0.1 CPM to 60 CPM in dioestrus (logFC of 8.8), and CXCL9, which increased from an average of 5 to 248 in oestrus (logFC of 5.7) and 5 to153 CPM in dioestrus (logFC of 4.6). Expression of the gene for CXCL11 increased from an average of 0 to 433 CPM in oestrus and from 0 to 205 CPM in dioestrus and that of the gene for CXCL16 increased from an average of 36 to 235 CPM in oestrus (logFC of 2.9) and from 17 to 108 CPM in dioestrus (logFC of 2.6).

*CX3CL1* was also significantly upregulated, increasing from 5 to 146 CPM in oestrus (logFC of 5.2) and from 2 to 36 CPM in dioestrus (logFC of 4.2).

The chemokine receptor gene with the highest levels of expression after inoculation with *E. coli* was that for CXCR7, with expression levels increasing from an average of 26 CPM to 270 in oestrus (logFC of 3.7) and 14 CPM to 57 CPM in dioestrus (logFC of 2.1). This also represented significantly higher levels of expression in oestrus 3 h post-inoculation than in dioestrus 3 h post-inoculation (logFC of 2.4). The gene for CXCR4 was expressed at its highest in oestrus pre-inoculation (92 CPM); significantly decreasing to levels of 29 CPM 3 h post inoculation (logFC of −1.1). In dioestrus, its expression increased slightly, but significantly from 9 CPM before inoculation to 25 CPM after inoculation of *E. coli* (logFC of 1.5).

#### Antimicrobial peptides and proteins

Genes for several antimicrobial peptides were significantly up-regulated 3 h after inoculation with *E. coli* (Fig. 2c). Expression of the equine beta defensin −1 (EBD1) gene increased from no copies before to an average of 49 (logFC of 9.5) after inoculation in oestrus and 44 CPM (logFC of 8.3) after inoculation in dioestrus. Similarly, expression of the gene for lysozyme showed a significant increase post-inoculation in both oestrus (logFC of 6.0) and dioestrus (logFC of 5.0). The gene for phospholipase sPLA_2_ was significantly up-regulated in oestrus following inoculation with bacteria, from pre-inoculation levels of an average of 2715 CPM to an average of 6271 CPM (logFC of 1.5), while copy numbers remained unchanged in dioestrus (1770 and 1752 CPM). Copy numbers after inoculation in oestrus were significantly higher than after inoculation in dioestrus.

The gene for SLPI (=eNAP-2) shows a significant increase in expression from pre-inoculation levels of 2 CPM to 152 CPM post-inoculation in oestrus (logFC of 6.7) and from 0.2 CPM pre-inoculation to 170 CPM post-inoculation in dioestrus (logFC of 9.4).

There were also significant increases in expression of the genes for lipocalin-2 and uteroferrin (ACP 5). Lipocalin-2 gene expression increased from 44 to 612 in oestrus (logFC of 4.1) and 22 CPM to 642 CPM in dioestrus (logFC of 3.8), while uteroferrin gene expression increased from pre-inoculation levels of 449 to 2008 post-inoculation in oestrus (logFC of 2.4) and from 389 CPM pre-inoculation to 2776 post-inoculation in dioestrus (logFC of 2.8). In contrast, there was only a significant increase in lactoferrin mRNA copy numbers in oestrus, from 118 to 342 CPM (logFC of 1.6), with the increase from an average of 42 to 65 CPM in dioestrus (logFC of 0.9) not found to be significant.

Low copy numbers (<20 CPM) of mRNAs for EBD-2, EBD-3, equine myeloid cathelicidin 3 (ECATH-3) and caspase-1 were detected post-inoculation, representing small but significant increases compared to pre-inoculation levels.

#### Matrix metallopeptidases

The expression of genes for several matrix metallopeptidases (MMP) associated with inflammation was also found to be up-regulated in response to intrauterine inoculation with *E. coli* (Fig. 2d). These included those for MMP1, which showed an increase in levels of expression from 0 to an average of 148 CPM in oestrus (logFC of 11.3) and from 0 to 53 CPM in dioestrus (logFC of 9.3). This represented a significant difference in expression between oestrus and dioestrus 3 h post-inoculation. Similarly, the genes for MMP3, MMP7 and MMP8 were significantly more highly expressed in oestrus than in dioestrus after inoculation with *E. coli. MMP3* mRNA levels increased from 5 to 162 CPM (logFC of 5.4) in oestrus and from 0 to 63 CPM in dioestrus (logFC of 11.1). *MMP7* mRNA levels increased from 0 to 33 CPM in oestrus (logFC of 7.0) and remained unchanged in dioestrus, while *MMP8* mRNA levels increased from 0 to 74 in oestrus (logFC of 10.2) and from 0 to 25 CPM in dioestrus (logFC of 8.4). This trend was also seen for the gene for MMP9, with expression levels increasing from 1 to 57 CPM in oestrus (logFC of 6.1) and 0 to 40 CPM in dioestrus (logFC of 6.2), but in this case the difference between cycle stages was not significant.

Finally, significant up-regulation of the gene for TIMP1, the tissue inhibitor of metalloproteinases 1, was also detected in both oestrus (logFC of 6.3) and dioestrus (logFC of 5.0) with levels of expression increasing from an average of 13 to 845 CPM in oestrus and from 8 to 272 CPM in dioestrus and expression log_2_ 2.1 higher in oestrus than in dioestrus after inoculation with *E. coli*.

## Discussion

High-throughput sequencing was used to evaluate the initial uterine response to the introduction of bacteria and to describe transcriptional changes associated with hormonal changes at two different oestrous cycle stages. Overall, nearly 3000 genes were found to be differentially expressed at significant levels in response to inoculation with *E. coli* in at least one cycle stage.

The effect of repeated endometrial biopsy collection was not evaluated in this study because it was assumed that these effects would be similar between cycle stages and outweighed by the response to the introduced bacteria. In addition, a recent study of the bovine liver has shown that repeated biopsies at 3 h intervals had no effect on hepatic cytokine gene expression [74].

As expected, most genes that were up-regulated 3 h after the inoculation of *E. coli* were associated with the innate immune response. Several areas were identified as being of particular interest and were investigated in more detail.

### Pathogen pattern recognition receptors

The first step in the host’s defence against a pathogen is detection of its presence by PRR, such as TLR or NLR [75]. As shown in Fig. 2a and b, the greatest up-regulation of gene expression in the TLR family after inoculation with *E. coli* was for *TLR2* and *4*.

TLR4 is a transmembrane molecule that recognizes lipopolysaccharides (LPS) produced by gram-negative bacteria [76, 77], explaining the need for its up-regulation in response to *E. coli.* A previous study in goats found that it was similarly up-regulated 3 h after inoculation [78]. Our study is the first to examine gene expression levels for *TLR2* and *4* in the equine endometrium after a challenge with bacteria and found a significant increase over time, but not between cycle stages. Previous studies on horses examining the response to frozen-thawed semen have yielded conflicting results 24 h after insemination [25, 79]. This ambiguity may be explained by the potential variation in the number of bacteria introduced into the uterus during insemination and also the later sample collection time used in this study. As PRRs are the host’s first line of detection and defence, stimulating the production of various cytokines, their up-regulation is likely to be most important in the initial hours after a challenge (Fig. 3).

Less is known about NLR expression in the equine endometrium. Within this family, we found the genes for NLRC4 and 5, and NLRP3 and 12, to show the greatest up-regulation in gene expression after the introduction of *E. coli* (Fig. 2b). Of particular interest was our observation that expression of several of these genes was significantly lower during dioestrus than during oestrus. This was seen for both the gene for the pro-inflammatory NLRP3, as well as for the gene for the regulatory molecules NLRC5 and 12. This is the first report of detection of *NLRC5* gene expression in the equine uterus, although it has been detected in the human uterus [34]. Of all the NLR genes detected in our study, *NLRC5* showed the greatest up-regulation after inoculation with *E. coli*. Interestingly, it has been implicated as an inhibitor of the production of pro-inflammatory cytokines, such as TNF, *IL*6, *and IL1B* [34, 35]. While the genes for some of these cytokines were up-regulated in our study, the transcription of *NLRC5* may be one of the initial steps limiting the inflammatory response to a physiological extent.

#### Cytokines

The response to the detection of PAMPs by PPR is the production of various pro-inflammatory cytokines by antigen-presenting cells (Fig. 3). As in earlier studies [24, 36], we found the genes for IL1B and IL8 to be significantly up-regulated 3 h after inoculation with *E. coli* in both oestrus and dioestrus. *IL*6 was up-regulated to a similar degree in dioestrus and oestrus, as has been seen previously in oestrus and after insemination [24, 80].

While we found that the TNF gene was expressed at significantly higher levels after inoculation of *E. coli*, the copy numbers were overall comparatively low (Fig. 5a). Previous studies in horses have detected peak expression levels at 3 h after inoculation of *E. coli* [24, 36, 78, 80]. These conflicting results might be due to different analysis methods or different gene sequence selection.

The anti-inflammatory cytokine genes for both IL10 and IL13 were expressed at very low levels and there was no significant increase in their expression 3 h after inoculation (Fig. 5b), while expression of genes for receptors for both these cytokines increased in response to *E. coli *infection (Figs. 4 and 5c). This may prepare the immune response for rapid down-regulation to prevent an excessive inflammatory response. However, only 3 h after exposure, expression of the ligand genes had not yet increased, potentially because the infection had not been contained. Christoffersen et al. detected a significant peak in *IL10* gene expression 3 h after inoculation with *E. coli* in dioestrus, but found no significant change during oestrus [24, 36]. Our study was similar to that of Christoffersen et al., making the difference in patterns of *IL10* gene expression more surprising. However, Christoffersen et al. analysed copy numbers of mRNAs relative to a housekeeping gene. Thus the level of expression of the *IL10* gene may be very low overall and a significant increase on this may still only result in relatively low copy numbers after inoculation. The analysis of later time points in future studies may potentially detect a greater increase in *IL10* expression after a longer period after inoculation.

The only gene for an anti-inflammatory cytokine showing large increases in expression 3 h after inoculation was that for IL1ra, whose expression was also a logFC of 1.97 higher in oestrus than in dioestrus at 3 h post inoculation with *E. coli*. IL1ra binds to IL1 receptors inhibiting the downstream effects of IL1α and IL1B [81]. The significant differences between oestrus and dioestrus 3 h after inoculation with *E. coli* may be explained by the greater numbers of *E. coli* found in the dioestrus samples than in the oestrus samples. This may suggest that the immune-regulatory cytokine IL1ra is expected to be expressed at higher levels in oestrus, when the infection is under more effective control 3 h post inoculation. In the earlier studies no significant increase was detected in *IL1ra* mRNA levels in healthy mares in response to inoculation with *E. coli* [24].

#### Chemokines

While several chemokines have been detected in the human uterus [82], with the exception of *CXCL8* (or *IL8*) little is known about their gene expression profile in the equine uterus during inflammation or in response to physiological changes [83].

Our study detected the presence and up-regulation of mRNAs for multiple chemokines in both the CCL and the CXCL families (Fig. 5d). The up-regulated expression of genes for chemokines detected after the inoculation of *E. coli* might result in recruitment of neutrophils, monocytes and T lymphocytes [84–89]. Interestingly, the gene for CCL2, which is a potent chemoattractant for monocytes and hence usually associated with chronic inflammation, was the second most highly expressed chemokine gene detected 3 h after inoculation with *E. coli* in our data [90, 91]. While the highest levels of expression might be expected for genes for neutrophil attractants at this early stage in the inflammatory process, several factors might contribute to the high levels of expression of the CCL2 gene. Transformation of monocytes into active macrophages may take longer than activation of neutrophils, CCL2 monomers need to form active multimers before being functional, and the CCL2 receptor needs to be stimulated before CCL2 has an effect [91]. Notably, CCR2, the receptor for CCL2, was only expressed at very low levels before and after inoculation (2–4 CPM), suggesting that the increased expression of the CCL2 gene was unlikely to have a chemotactic effect on the inflammatory response in the uterus at this early stage.

Another group of chemokine genes that were upregulated were those for the CXC ligands 9–11, which mainly attract T lymphocytes of the adaptive immune system. The expression of the *CXCL10* gene was particularly greatly increased in response to infection with *E. coli* (Fig. 5d). It is possible that, rather than its chemotactic functions, its documented anti-microbial function may be more relevant at this point in the immune response [92, 93]. The same explanation may also account for the increase in *CCL20* gene expression [93].

#### Antimicrobial peptides and proteins

Gene expression profiles for a large number of other antimicrobial immune molecules were also assessed in the equine uterus in our study (Fig. 2c). The expression of several of these was induced by the inoculation of *E. coli*. This correlation with induced endometritis has previously been described for lysozyme in response to an intrauterine infusion of *Sc. zooepidemicus* [94]. Increased levels of *sPLA*_*2*_ mRNA have only been detected in association with induced abortion before [95], while our study found similar mRNA levels at the two stages of the oestrous cycle, but a significant increase in response to the intrauterine inoculation of *E. coli* only during oestrus (Fig. 2c). The three antimicrobial peptides, EBD1, lysozyme and sPLA_2_, can interfere with the permeability of bacterial cell walls and the genes for all three were more highly expressed after inoculation with *E. coli* in oestrus than in dioestrus, even though the difference was only significant for the *sPLA*_*2*_ gene (Fig. 2c). This may have contributed to the quicker elimination of *E. coli* during oestrus seen in our experiment.

The selective bacterial enzyme inhibitor SLPI has previously been described in the equine uterus as eNAP-2 [96]. Expression of the gene encoding it was significantly increased in both oestrus and dioestrus after inoculation with *E. coli* with the inter-sample variation being much greater in dioestrus. The oestrous uterus may react to the introduction of bacteria with a more regulated response, while a less controlled response is seen to the unexpected introduction of bacteria in dioestrus.

Kolm et al. analysed lactoferrin gene expression in mares resistant and susceptible to PMIE throughout the oestrous cycle [97] and, as in our study, detected significantly higher levels of expression in oestrus than dioestrus. While levels of expression of the lactoferrin gene remained at pre-inoculation levels after exposure to *E. coli* in dioestrus, there was a significant increase post-inoculation in the oestrus uterus. Mares with signs of chronic endometritis show increased levels of lactoferrin gene expression throughout the cycle compared to resistant mares [97]. These observations, in combination with our own, suggest that lactoferrin may play a role in both the pathogenesis and the treatment of PMIE and that hormone levels may have a strong influence on its expression.

Lipocalin-2 and uteroferrin have previously only been described in the equine uterus in association with pregnancy [47, 98]. However, the significant increases in expression of the genes encoding these factors in response to *E. coli* in both oestrus and dioestrus suggest that they play a significant role in the uterine innate immune response as well.

Finally, we identified expression of several antimicrobial-associated genes not previously detected in the equine uterus. These include the genes for the beta-defensins EBD2 and EBD3, the cathelicidin ECATH-3, caspase-1 and NK-lysin. While they did not appear to be directly involved in the acute response to bacterial endometritis caused by *E. coli*, they may still be important in other aspects of the uterine immune response.

Overall, our data suggests that antimicrobial peptides may have received less attention than they deserve as potential treatment targets for equine endometritis.

#### Matrix metallopeptidases and their immune function

MMPs have complex interactions with the immune system, both activating and degrading chemotactic molecules [48]. However, most previous studies have been done *in vitro,* in murine knock-out models or in humans, and only *MMP2* and *9* have been detected in the equine uterus in association with inflammatory disease [51]. In our study, *MMP9* gene expression was significantly up-regulated after inoculation of *E. coli* (Fig. 2d) without a significant difference between oestrus and dioestrus 3 h after inoculation of *E. coli*, while Oddsdottir et al. detected significantly higher levels of *MM9* gene expression 5 h after inoculation of *Sc. zooepidemicus* in dioestrus compared to the levels seen in oestrus at the same time point [51]. MMP9 can enhance the chemotactic activity of IL8 [99, 100], the gene for which was expressed at significantly higher levels post-inoculation in our study. MMP8, the gene for which levels were significantly higher in oestrus after inoculation than in dioestrus after inoculation (Fig. 2d), has also been shown to activate IL8 [101]. MMP7, the gene which was only up-regulated in oestrus in response to *E. coli* (Fig. 2d), is also indirectly associated with neutrophil chemotaxis by activating CXCL1 [102]. Our data suggest that there is significant redundancy in the uterine innate immune response, potentially ensuring rapid and effective neutrophil migration.

*MMP1* and *3*, the genes for which were expressed at significantly higher levels 3 h after inoculation in oestrus and dioestrus (Fig. 2d), have a more regulatory function, inactivating CCL2, IL8 and CCL13 [103]. In addition, the gene for TIMP1, an inhibitor of most MMPs, was expressed at higher levels post-inoculation than any of the MMP in our study. This might suggest that, while the chemotactic function is still required 3 h post-inoculation to attract neutrophils, the uterus is already preparing itself to terminate inflammatory signals as soon as bacterial invasion is under control.

Interestingly, expression of most MMP genes was greater after inoculation in oestrus than in dioestrus (Fig. 2d). In a previous study, we found that matrix remodeling was one of the main pathways with higher levels of expression in oestrus compared to dioestrus 5 days after ovulation [73]. However, only the *MMP3* gene was already expressed at different levels in oestrus and dioestrus prior to inoculation. Therefore it seems likely that up-regulation of expression after inoculation of *E. coli* was a response to bacterial infection rather than to changes in hormone levels. However, the very high level of expression of the *TIMP1* gene in oestrus suggests that the initial innate immune response may already be starting to slow down 3 h after inoculation.

## Conclusion

*E. coli*, the bacteria most commonly isolated from mares with fertility problems [11], have a generation time of around 20 min; thus, a rapid immune response is essential for containment of infection. However, an excessive or prolonged inflammatory response will damage tissue integrity and, in the uterus, also interfere with successful pregnancy.

While the reduced uterine clearance observed in dioestrus may be partly attributable to the closure of the cervix and reduced myometrial contractility in dioestrus, our study has clearly demonstrated that there are also significant differences in gene expression (Fig. 1). These changes may generate a uterine environment in dioestrus that is similar to that seen in oestrus in mares susceptible to PMIE. In future studies, evaluation of expression after the first 3 h in dioestrus and in mares susceptible to PMIE may provide valuable additional insights into the effect of the cycle stage on the duration of the inflammatory process.

Our observations emphasize the complexity of interactions in the immune response and the tight balance between multiple pro- and anti-inflammatory factors required for rapid and efficient elimination of bacteria. However, while we have identified a large number of genes of potential interest, future studies will need to examine protein expression.

This study is one of the first high-throughput analyses of the uterine inflammatory response in any species and can potentially be used to model acute endometritis in other species, including humans.

## Availability of supporting data

The raw RNA sequence data have been deposited in the NCBI’s Sequence Read Archive (Bioproject ID: PRJNA270116; http://www.ncbi.nlm.nih.gov/bioproject/PRJNA270116/).

## Additional files

Additional file 1: Figure S1. Flow chart outlining the study design, time points of *E. coli* inoculations and collection of uterine samples. Three horses were assigned to group 1 and two to group 2. (PDF 12 kb) Additional file 2: Figure S2. Heatmap of all genes analysed for significant differential expression between the two cycle stages before and 3 h after inoculation with *E. coli*. Each column represents one sample, while each row represents the log2 counts per million (CPM) of one gene with normalised standard scores (Z-expression values; −2/ light green to +2/ dark green). The cluster trees on the top and left margins represent the hierarchical relation between samples and gene counts, respectively. While the first letter stands for the horse, the second stands for (o)estrus (E) or dioestrus (D), 0 h stands for samples taken before inoculation and 3 h for samples taken 3 h thereafter. Note that biological replicates cluster according to their treatment with the exception of ED_3h, which clusters with the dioestrous samples before inoculation. (PNG 985 kb)

## Abbreviations

CCL and CXCL: Chemokine families
CFU: Colony forming units
CPM: Copies per million reads
*E. coli*: *Escherichia coli*
EBD1: Equine beta defensin −1
GO: Gene ontology (GO:BP – biological process; GO:MF – gene ontology molecular function; GO:CC – cellular component)
IL: Interleukin
KEGG: Kyoto encyclopaedia of genes and genomes
logFC: Log_2_ fold change
MMP: Matrix metallopeptidases
NLR: NOD-like receptor
PMIE: Persistent mating-induced endometritis
PRR: Pattern recognition receptor
SLPI: Secretory leukoprotease inhibitor (also eNAP-2)
sPLA_2_: Secreted phospholipase A_2_
TLR: Toll-like receptor
TNF: Tumor necrosis factor
