# Supplementary material for: Deep sequencing of the uterine immune response to bacteria during the equine oestrous cycle
Source: BMC Genomics. 2015 Nov 14;16:934. doi: 10.1186/s12864-015-2139-3 (PMC4647707; doi:10.1186/s12864-015-2139-3)
Supplement: Additional file 1: Figure S1. — Flow chart outlining the study design, time points of E. coli inoculations and collection of uterine samples. Three horses were assigned to group 1 and two to group 2. (PDF 12 kb) [file 12864_2015_2139_MOESM1_ESM.pdf]

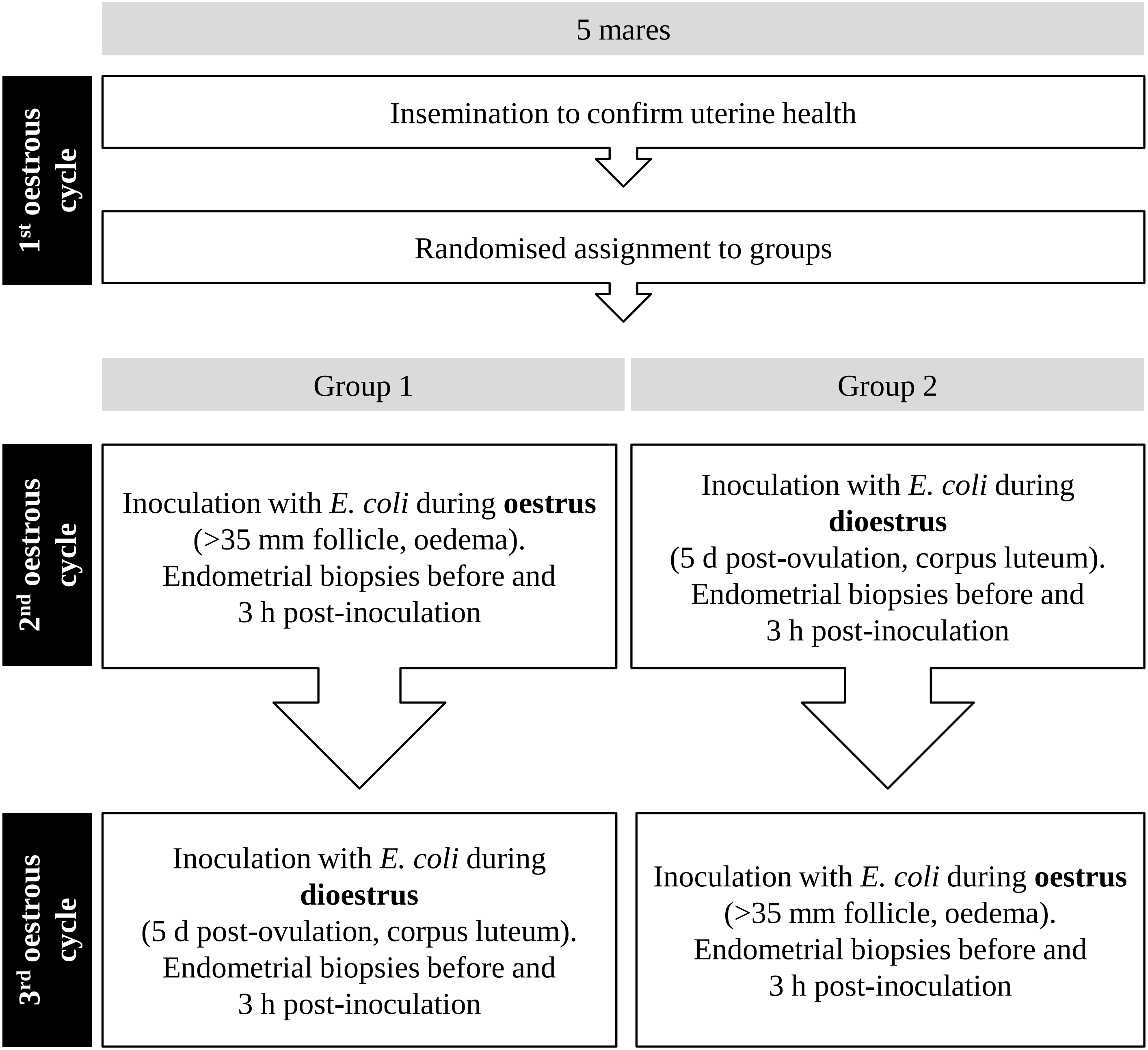
5 mares
1st oestrous cycle
Insemination to confirm uterine health
Randomised assignment to groups
Group 1
Group 2
2nd oestrous cycle
Inoculation with *E. coli* during **oestrus** (>35 mm follicle, oedema). Endometrial biopsies before and 3 h post-inoculation
Inoculation with *E. coli* during **dioestrus** (5 d post-ovulation, corpus luteum). Endometrial biopsies before and 3 h post-inoculation
3rd oestrous cycle
Inoculation with *E. coli* during **dioestrus** (5 d post-ovulation, corpus luteum). Endometrial biopsies before and 3 h post-inoculation
Inoculation with *E. coli* during **oestrus** (>35 mm follicle, oedema). Endometrial biopsies before and 3 h post-inoculation
