# Supplementary figures and images for: Deep sequencing of the uterine immune response to bacteria during the equine oestrous cycle
Source: BMC Genomics. 2015 Nov 14;16:934. doi: 10.1186/s12864-015-2139-3 (PMC4647707; doi:10.1186/s12864-015-2139-3)

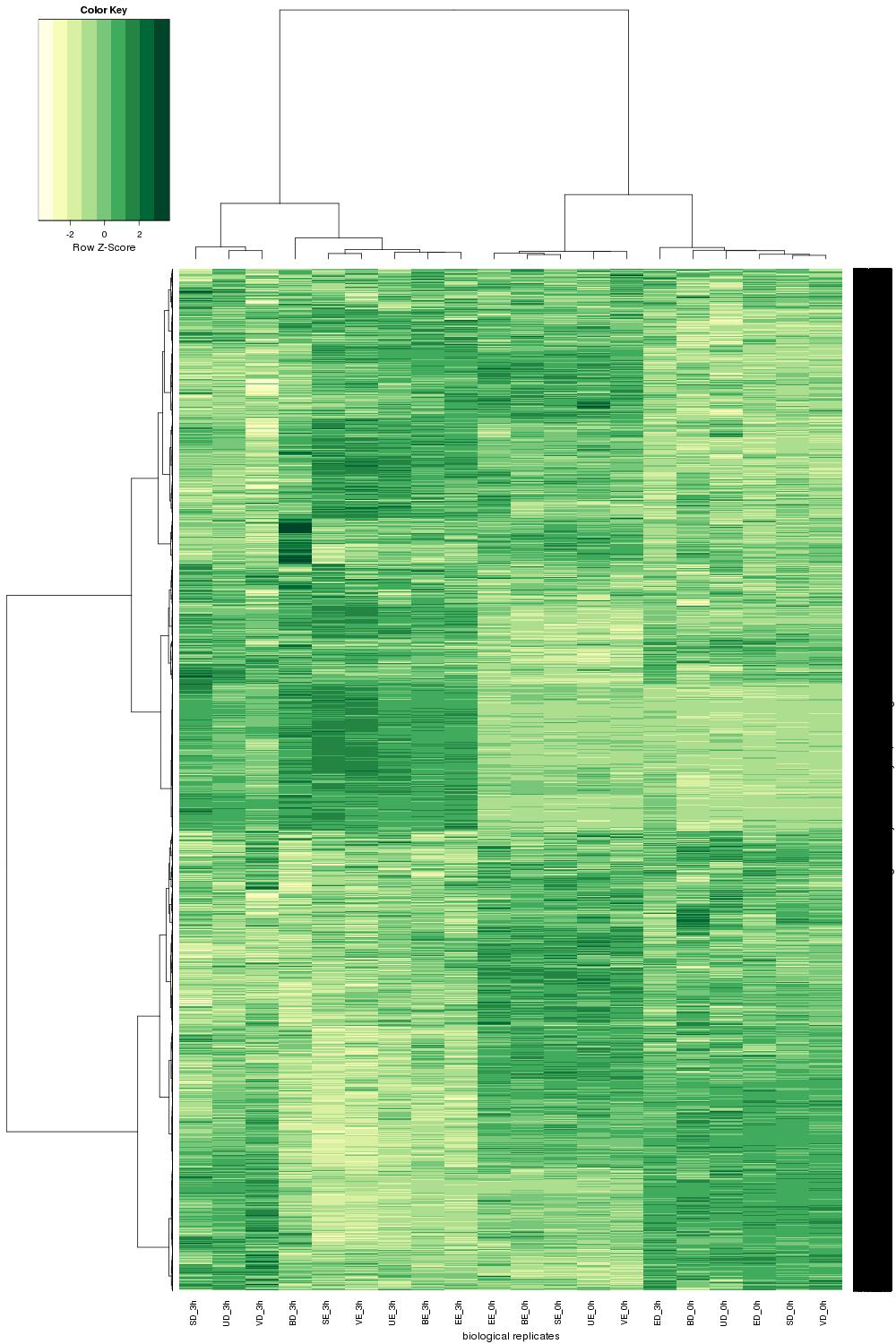

Supplement: Additional file 2: Figure S2. — Heatmap of all genes analysed for significant differential expression between the two cycle stages before and 3 h after inoculation with E. coli. Each column represents one sample, while each row represents the log2 counts per million (CPM) of one gene with normalised standard scores (Z-expression values; −2/ light green to +2/ dark green). The cluster trees on the top and left margins represent the hierarchical relation between samples and gene counts, respectively. While the first letter stands for the horse, the second stands for (o)estrus (E) or dioestrus (D), 0 h stands for samples taken before inoculation and 3 h for samples taken 3 h thereafter. Note that biological replicates cluster according to their treatment with the exception of ED_3h, which clusters with the dioestrous samples before inoculation. (PNG 985 kb) [file 12864_2015_2139_MOESM2_ESM.png]
